# Supplementary material for: Injectable bioresorbable conductive hydrogels for multimodal brain tumor electroimmunotherapy
Source: Nat Commun. 2025 Nov 3;16:9702. doi: 10.1038/s41467-025-65785-x (PMC12583519; doi:10.1038/s41467-025-65785-x)
Supplement: Supplementary file 1 — Supplementary Information [file 41467_2025_65785_MOESM1_ESM.pdf]

Injectable Bioresorbable Conductive Hydrogels for Multimodal Brain Tumor Electroimmunotherapy

Authors: Amit Singh Yadav[1], Umut Aydemir[1], Karin Hellman[1], Peter Ekström[1], Abdelrazek H. Mousa[2,3], Jiaxin Li[4], Muhammad Anwar Shameem[2], Cedric Dicko[5], Johan Bengzon[4,6], Fredrik Ek[1], Martin Hjort[1] and Roger Olsson[1,2,7]*

[1]Chemical Biology & Therapeutics, Department of Experimental Medical Science, Lund University; SE-221 84 Lund, Sweden,

[2]Department of Chemistry and Molecular Biology, University of Gothenburg; SE-405 30 Gothenburg, Sweden.

[3]Chemistry Department, King Fahd University of Petroleum and Minerals; Dhahran 31261, Saudi Arabia

[4]Department of Neurosurgery, Skane University Hospital; Lund, Sweden

[5]Pure and Applied Biochemistry, Department of Chemistry, Lund University, Lund, Sweden

[6]Kamprad Laboratory, Department of Clinical Sciences, Lund University; Lund, Sweden

[7]Department of Chemistry, Lund University; P.O. Box 124, 221 00 Lund, Sweden

*Corresponding author. Email: roger.olsson@med.lu.se

Supplementary methods

In vitro erosion of A5:ETE-PC electrode

A5:ETE-PC (20:40 mg ml$^{-1)}$ was added on agarose gel (0.5% in Ringer's solution) and left to diffuse for 2 min, followed by electrofunctionalization (EF) for various durations (0–5 min). After the EF, the agarose blocks with A5:ETE-PC were submerged in PBS (pH 7.4) and kept on mild shaking at 37 °C. The PBS was replaced with fresh PBS every day. The erosion was monitored by phase contrast imaging at various time points (0–14 days) using a 4x objective on Olympus BX53 microscope.

Macrophage preparation and A5:ETE-PC treatment

Primary human blood leukocyte concentrate was obtained from Laboratoriemedicin at Skåne University Hospital (Lund, Sweden). The sample was filtered through a 70 µm cell strainer into a 50 mL Falcon® tube and diluted 1:1 with DMEM supplemented with 10% fetal bovine serum (FBS). To separate peripheral blood mononuclear cells (PBMCs) containing $CD14^+$ monocytes, the diluted suspension was carefully layered onto 15 mL of LymphoPrep™ (STEMCELL Technologies) in a 50 mL tube and centrifuged at 800 × *g* for 20 minutes at room temperature. After centrifugation, the buffy coat was collected slowly to avoid disturbing the gradient, diluted 1:1 with DMEM + 10% FBS, and centrifuged at 500 × *g* for 10 minutes. The resulting pellet was resuspended in CD34+ buffer (PBS without Ca^{2+}/Mg^{2+}, 0.4% EDTA, and 5% heat-inactivated FBS), and cells were counted using a Bürker chamber. Cells were then pelleted again (350 × *g*, 7 minutes, room temperature) and resuspended in 80 µL of CD34+ buffer per 10×10^6 cells. CD14 MicroBeads (Miltenyi Biotec®, 20 µL per 10×10^6 cells) were added, and the suspension was incubated on ice for 15 minutes with gentle agitation. Following incubation, the cells were washed with CD34+ buffer and applied to a pre-washed magnetized column mounted in a magnetic separator. $CD14^+$ monocytes were retained in the column, while unlabeled lymphocytes (T cells, B cells, and natural killer cells) were discarded. The column was then removed from the magnet, and the positively selected $CD14^+$ monocytes were eluted and counted for further processing.

$CD14^+$ monocytes were seeded in 6-well plates at a density of 5×10^5 cells per well. After 24 hours of adherence, the cells were stimulated with 5 ng/mL recombinant human GM-CSF (20 µg/mL stock solution,) and cultured for 48 hours at 37 °C in a humidified atmosphere containing 5% CO_2. Cells were maintained in RPMI 1640 medium (Gibco) supplemented with 10% fetal bovine serum (FBS; Gibco), 100 U/mL penicillin, 100 µg/mL streptomycin, and 1% non-essential amino acids. The differentiation into macrophages was monitored by assessing morphological changes under a light microscope. Following morphological assessment to confirm differentiation, macrophages were treated with 1 mg/mL of ETE-PC/A5 solution (The stock solution was prepared at a concentration of 40 mg/mL in sterile, indicator-free complete DMEM, electrofunctionalized in vitro, and subsequently diluted to 1 mg/mL). Prior to application, the solution was filtered through a 40 µm cell strainer (pluriStrainer). Cells were incubated with the ETE-PC/A5 treatment for 48 hours at 37 °C in a 5% CO_2 atmosphere. Morphological responses were monitored by light microscopy after the treatment period.

Mechnical testing of A5:ETE-PC drape covered tumor cavities

A Biomomentum Mach-1 mechanical tester was used to deduce the mechanincal properties of A5:ETE-PC drape covered tumor cavities. We employed a similar method as describe previously[1,2]. Tumors with resection cavities were coated with A5:ETE-PC, electrofunctionalized at 1.2V for 2.5min, left for ETE-PC diffusion for 5min, and electrofunctionalized at 3V for 5min. Prior to experiments, the tumors were removed from the CAM and embedded in agarose for ease of handling. Indenter approached the tumor cavities from atop.

The test was performed in indentation mode, with a 500µm diameter spherical indenter at indentation depths of 100, 200, and 300µm. The test profile consisted of the following steps:

- Contact. Contact force of 0.05gf, contact rate of 0.02 mm/s
- 10 min wait to recover from contact
- 3 stress-relaxations at 100µm, 200µm, and 300µm depth with a ramp time of 0.05mm/s
- 4 sinusoidal test at 0.1, 1, 1.4 and 2Hz

The data (shear moduli and relaxation times) were analyzed following Wang et al and Babei et al[3,4]. The elastic (Young) moduli were estimated from the contact trace using a Hertzian model (Biomomentum built-in analysis software).

Supplementary references

1. Hjort, M. et al. In situ assembly of bioresorbable organic bioelectronics in the brain. *Nat. Commun*. **14**, 4453 (2023).

2. Aydemir, U. et al. In situ assembly of an injectable cardiac stimulator. *Nat. Commun*. **15**, 6774 (2024).

3. Wang, M. et al. Characterizing poroelasticity of biological tissues by spherical indentation: an improved theory for large relaxation. *J. Mech. Phys. Solids.* **138**, 103920 (2020).

4. Babaei, B., Davarian, A., Pryse, K.M., Elson, E.L. and Genin, G.M. Efficient and optimized identification of generalized Maxwell viscoelastic relaxation spectra. *J. Mech. Behav. Biomed. Mater.* **55**, 32-41 (2016).

Supplementary Figures

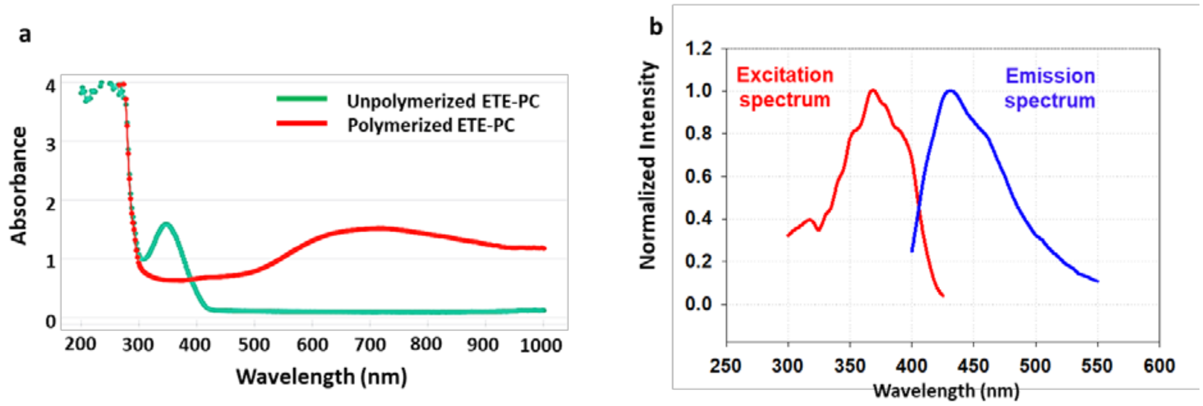

Fig. 1: Physicochemical characterization of ETE-PC (a) UV-Visible spectra of unpolymerized vs polymerized ETE-PC. **(b)** Fluorescence spectra of ETE-PC representing excitation and emission maxima.

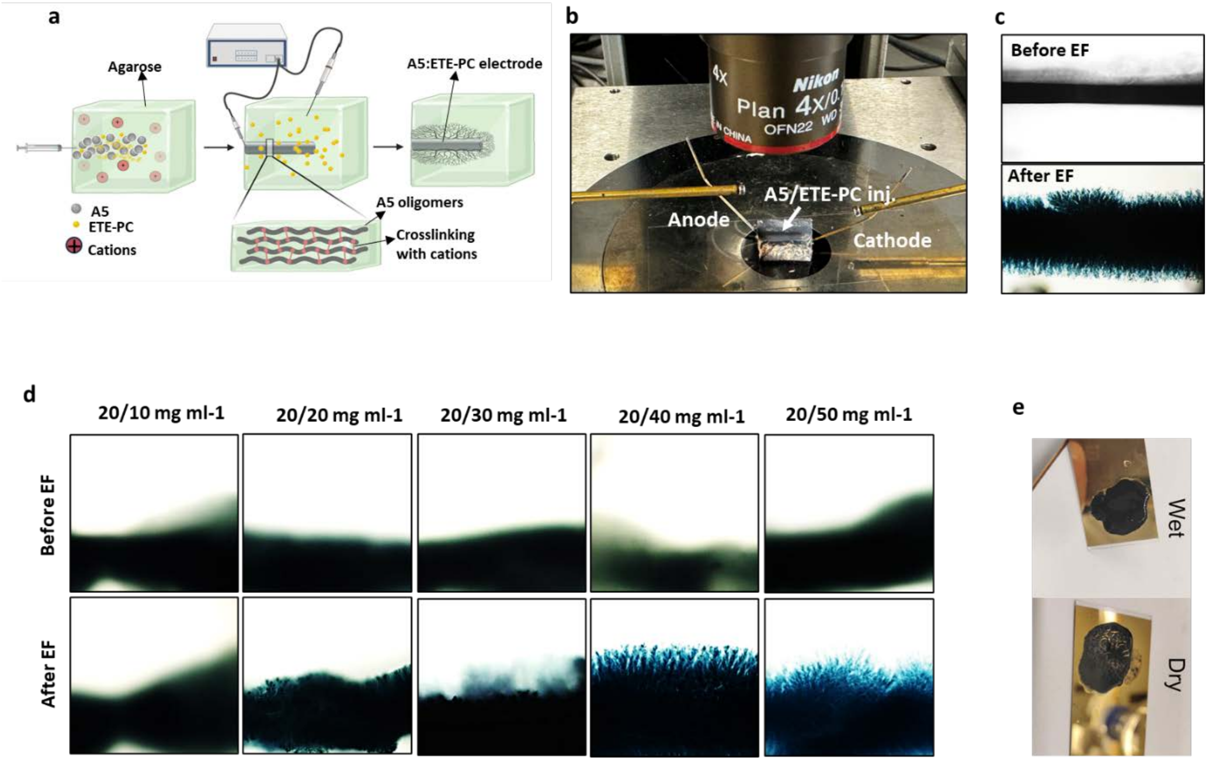

Fig. 2: Evaluation of A5:ETE-PC 3D flexible electrode in agarose. (a) Schematic representation of A5:ETE-PC electrode assembly in agarose. **(b)** Experimental setup for electrofunctionalization of ETE-PC on A5 for electrode assembly after injection in agarose mold. **(c)** ETE-PC electrofunctionalization on A5 after applying a bias of 2V via Au-coated W-electrode; n=3. **(d)** Effect of ETE-PC concentration on dendrite formation, n=1. **(e)** A “bulk hydrogel by electrofunctionalizing A5:ETE-PC on an Au-coated glass slide. The gel was weighed before and after drying at 100°C to examine water content. **Figure 2a** created with BioRender.com released under a Creative Commons Attribution-NonCommcercial-NoDerivs 4.0 International license (https://BioRender.com/8864ndt).

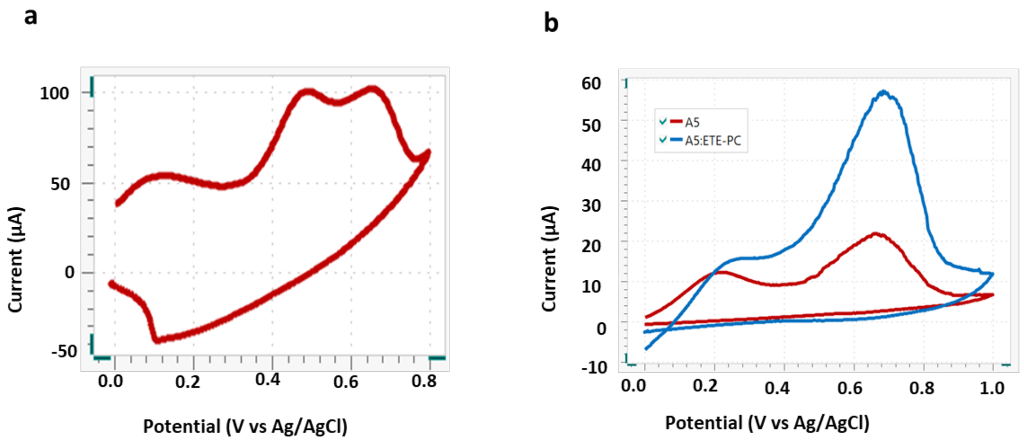

Fig. 3: (a) Cyclic voltammetry characterization of ETE-PC electrofunctionalization on A5 recorded between –0.01 and 0.8 V potential range and 10 mV/ s scan rate in 0.5% agarose (PBS, pH7.4). **(b)** A5 vs. A5:ETE-PC Cyclic voltammograms recorded between –0.01 and 0.8 V potential range and 50 mV/s scan rate in 0.5% agarose (PBS, pH7.4).

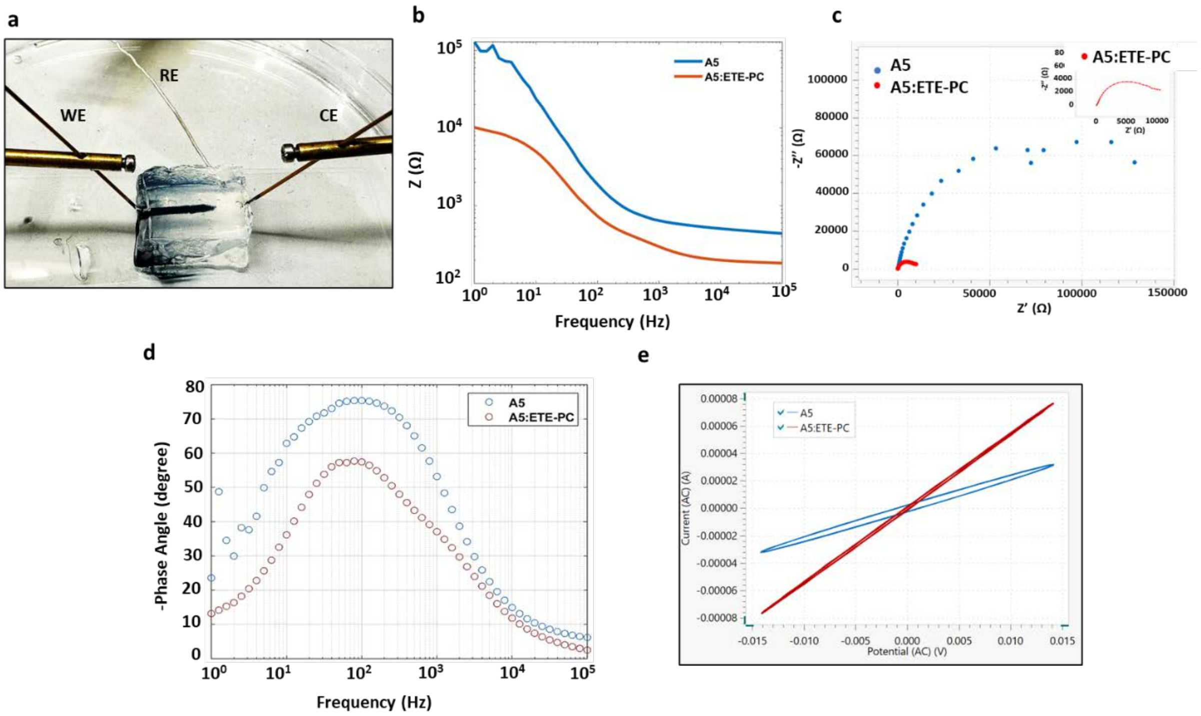

Fig. 4: Electrical impedance spectroscopic evaluation of A5 vs A5:ETE-PC electrode. (a) Three electrode system including A5:ETE-PC electrode (externally connected with Au coated metal electrode) as working electrode, Au coated metal as counter electrode and Ag/AgCl as reference electrode for electrochemical impedance spectroscopy (EIS) analysis in agarose mold. **(b)** EIS characterization is used to compare the impedance of Au vs. A5:ETE-PC electrode in 0.5% agarose. Bode plot demonstrate impedance. **(c)** Nyquist plots of EIS measurement using A5 or A5:ETE-PC electrodes in agarose mold (inset: magnified Nyquist plot for A5:ETE-PC electrode). **(d)** Bode plot of EIS measurement using A5 or A5:ETE-PC electrodes in agarose mold showing phase angle. **(e)** Lissajous plot showing current as a function of potential for EIS measurements using A5 or A5:ETE-PC electrodes in agarose mold.

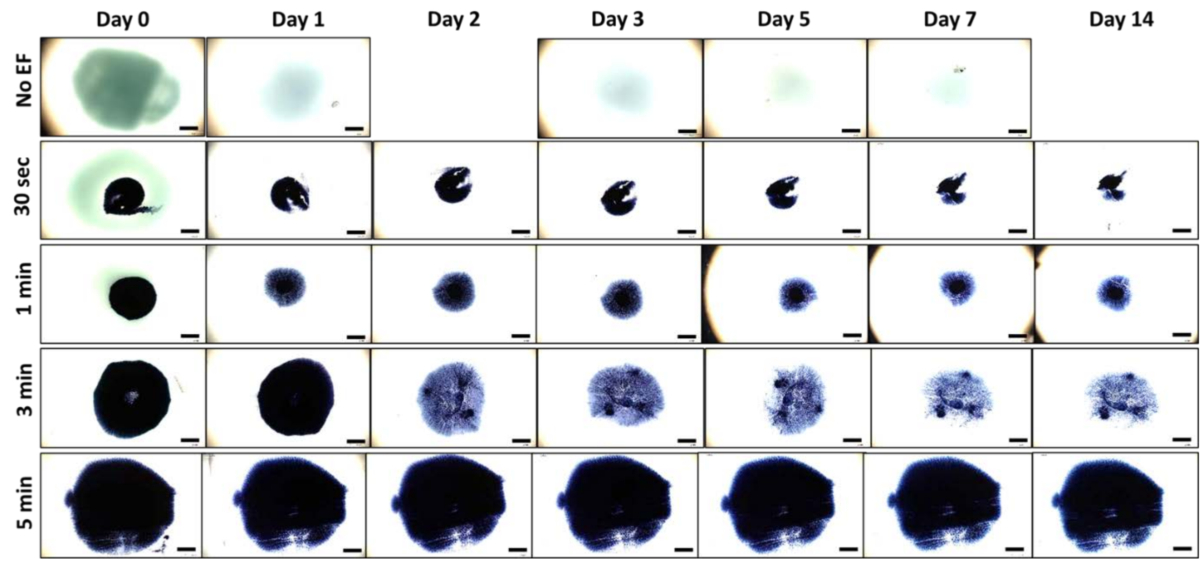

Figure 5. Erosion of A5:ETE-PC in physiological buffer (PBS). A5:ETE-PC (20:40 mg ml^{-1}) was added on agarose gel (0.5% in Ringer's solution) and left to diffuse for 2 min, followed by electrofunctionalization (EF) for various durations (0–5 min). After the EF, the agarose blocks with A5:ETE-PC were submerged in PBS (pH 7.4) and kept on mild shaking at 37 °C. The erosion was monitored by imaging at various time points (0–14 days). Scale bar: 500 µm.

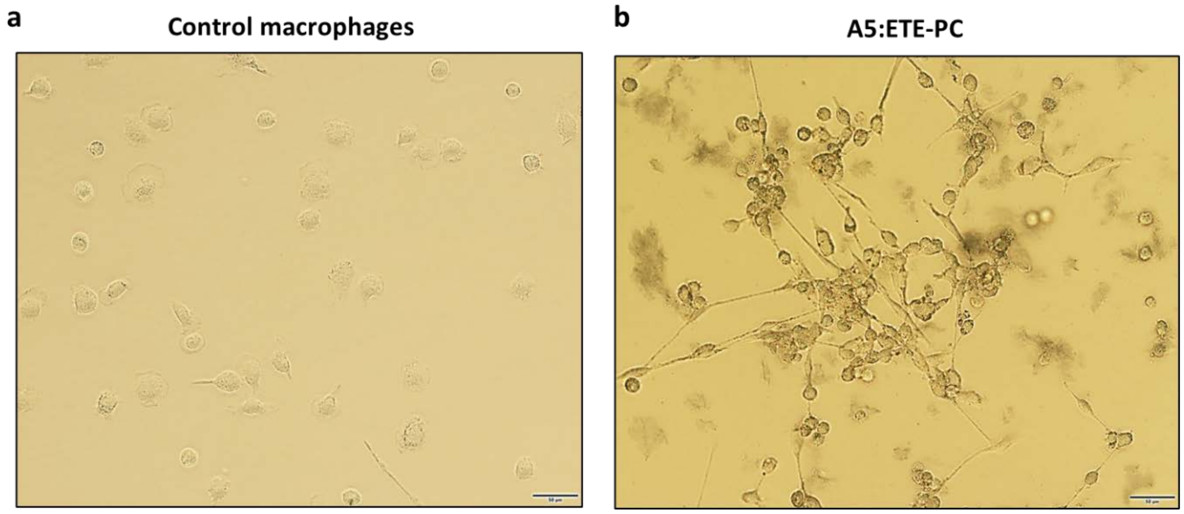

Figure 6. Morphological evaluation of macrophages following ETE-PC/A5 treatment. light microscopy images of macrophages after 48 hours in culture. **(a)** Untreated control macrophages displaying typical spread morphology and clear cytoplasm. **(b)** Macrophages treated with 1 mg ml^{-1} A5:ETE-PC solution exhibit altered morphology and darker cytoplasm due to material interaction and engulfment. Scale bar: 50 µm.

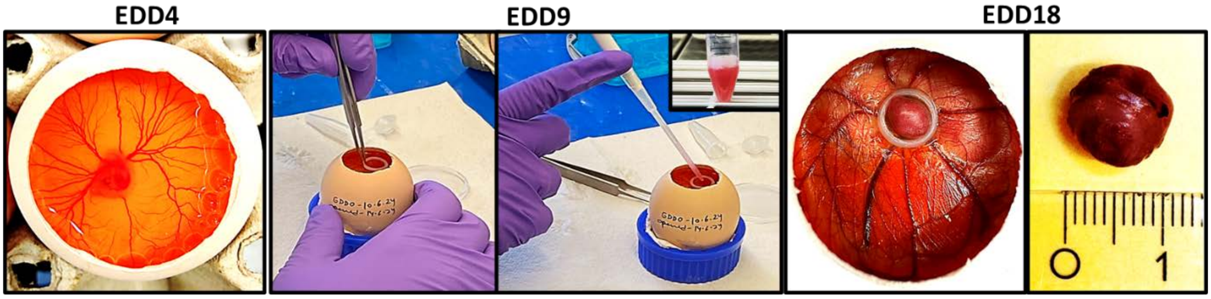

Fig. 7: Development of chicken chorioallantoic membrane (CAM) in vivo tumor model.

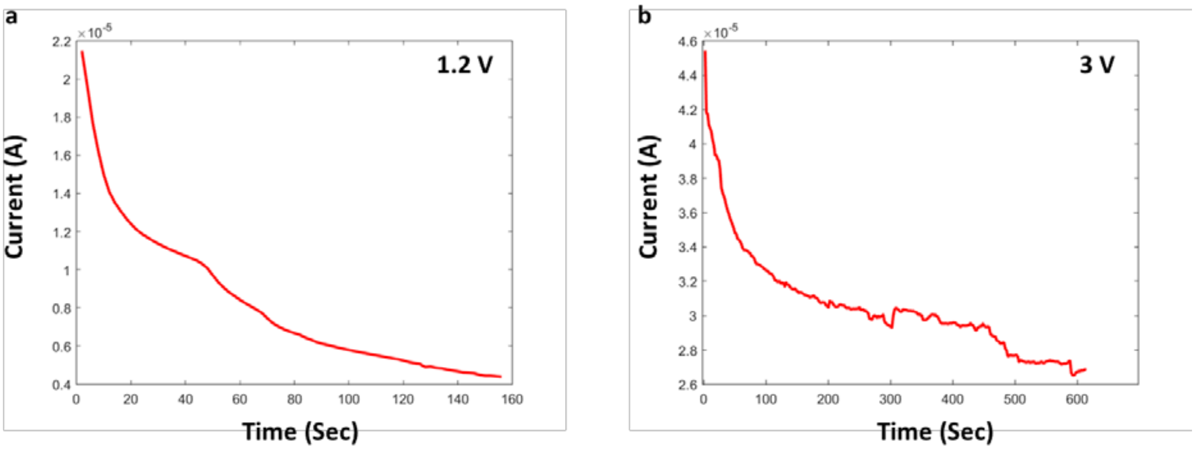

Fig. 8: Evaluation of A5:ETE-PC electrode in U87 tumors. Current flow during *in vivo* electrofunctionalization of A5+ETE-PC, **(a)** 1.2 V was initially applied for 2.5 minutes between the microcapillary and counter electrode during functionalization **(b)** Second electrofunctionaliztion was performed after 5 min by applying 3V for 10 min.

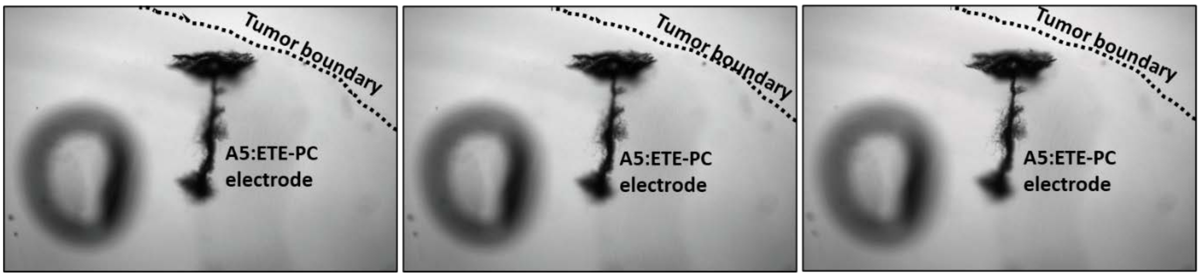

Fig. 9: 3D reconstructed image of A5:ETE-PC electrode in tumor at various focuses after tissue clearing process.

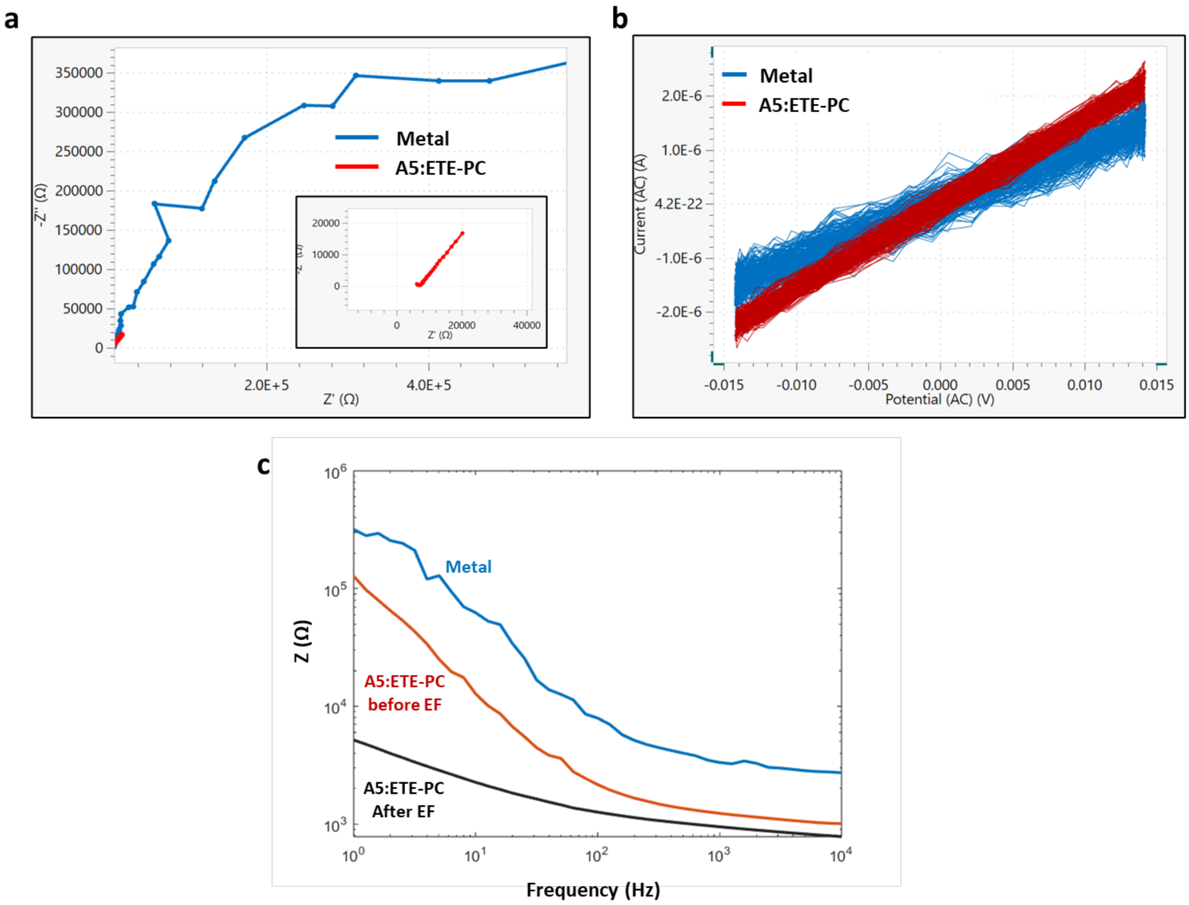

Fig. 10: Electrochemical impedance spectroscopic evaluation of A5:ETE-PC electrode in U87 tumors. (a) Nyquist plots of EIS measurement with metal or A5:ETE-PC electrode in tumor. (inset: magnified Nyquist plot for A5:ETE-PC electrode in tumor). **(b)** Lissajous plot showing current as a function of potential for EIS measurements with metal or A5:ETE-PC electrode in tumor. **(c)** EIS characterization was performed to examine impedance in the tumor using metal (Pt-Pd coated capillary) or A5:ETE-PC electrode before and after electrofunctionalization.

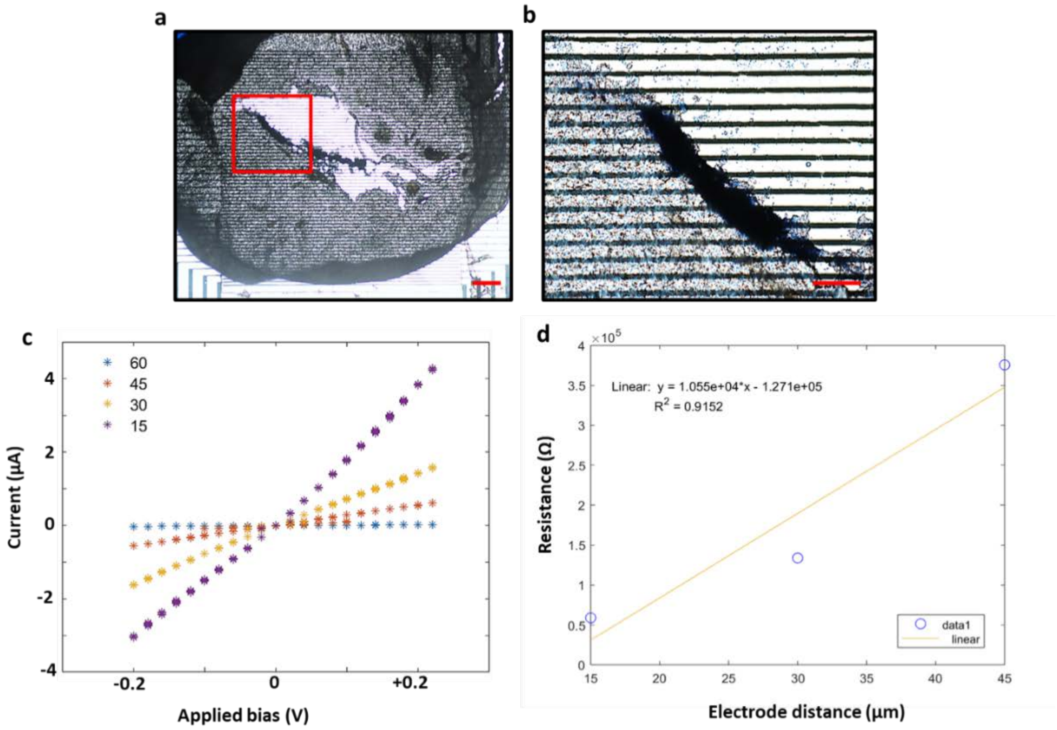

Fig. 11: Electrical properties of A5:ETE-PC in tumor tissue. (a-c) Electrical measurements of sectioned tumor injected with A5:ETE-PC. Images show lower and higher magnification of A5:ETE-PC containing tumor section on glass slide with interdigitated gold electrodes. Current–voltage sweeps obtained with increasing distance between electrodes; scale bar: 300 µm (a), 100 µm (b). **(d)** Linear Curve Fitting of resistance vs electrode distance data acquired from electrical measurements of A5:ETE-PC containing tumor section on glass slide with interdigitated gold electrodes. Data point at 60 µm was excluded as it was out of range.

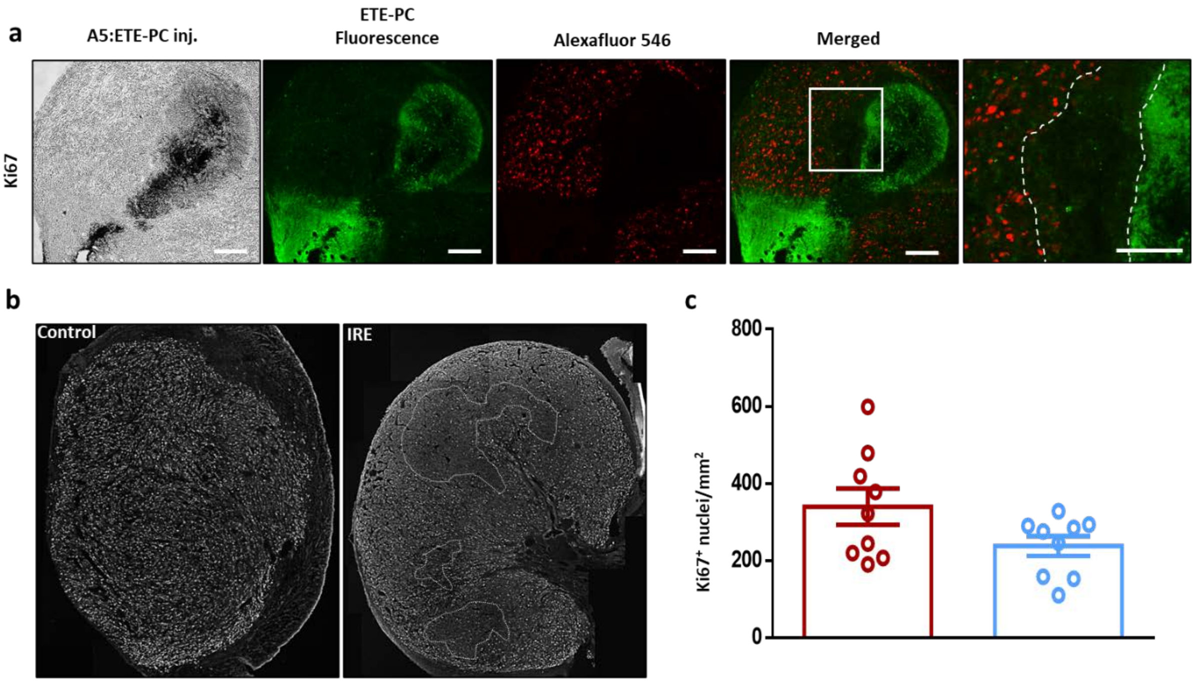

Fig. 12: A5:ETE-PC electrode mediated IRE inhibits tumor cell proliferation. (**a**) Fluorescence images of IRE-treated tumor (3x10, 1000 V pulses, 1ms) demonstrate staining of Ki67 in U87 tumor sections (16 µm). ETE-PC monomer fluorescence (green), Ki67 (red). Scale bar: 200 µm. n=2 independent experiments **(b)** Ki67 stained nuclei in binary images of the whole tumor section created from multiple overlapping images. **(c)** The number of $Ki67^{+}$ nuclei and the area of the whole section were calculated from 9 different sections in control and IRE groups each. The plot represents $Ki67^{+}$ nuclei/mm^2 in tumor sections, mean ± SEM, n=9 from 3 independent experiments.

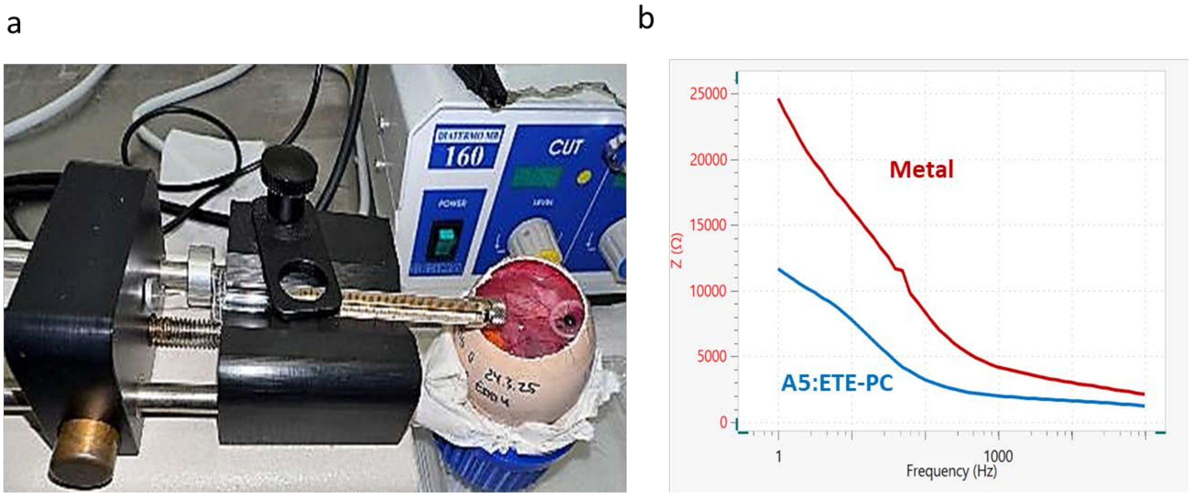

Fig. 13: Convection enhanced delivery of A5: ETE-PC in U87 tumor. (**a**) Set up for convection enhanced delivery of A5:ETE-PC in U87 tumor (**b**) Bode plot showing impedance of metal vs A5:ETE-PC electrode injected via CED.

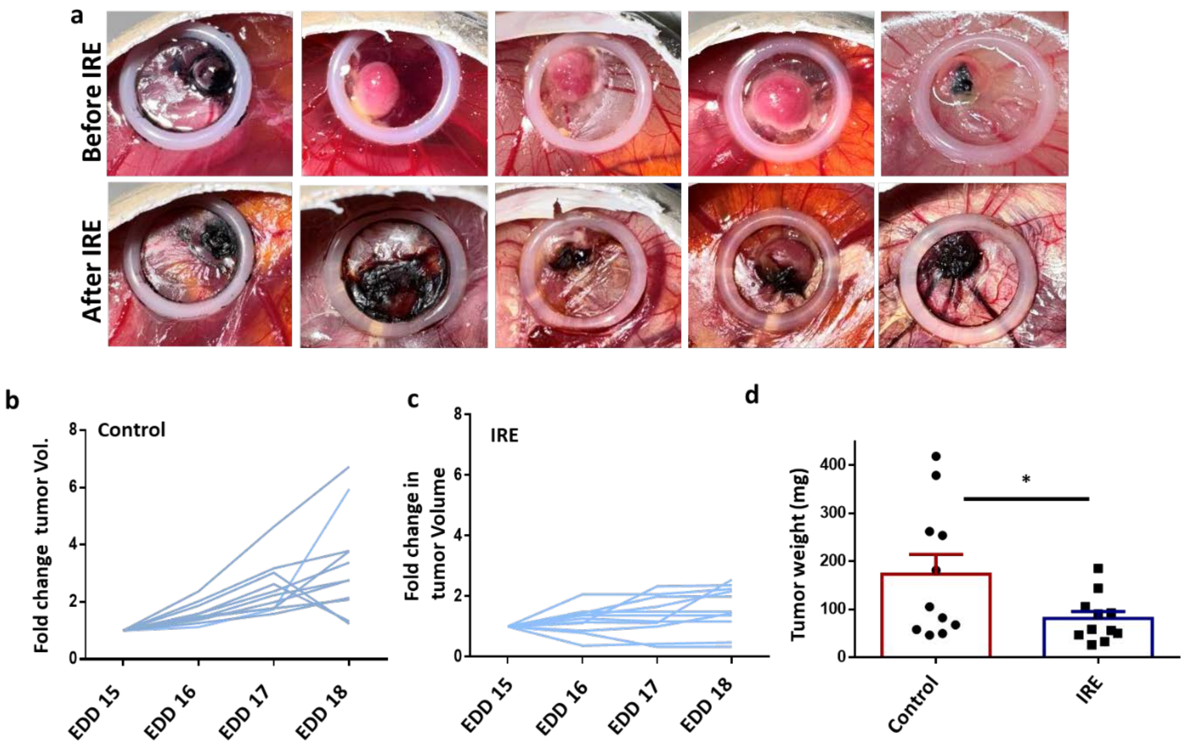

Fig. 14: IRE via A5: ETE-PC electrode abrogates U87 tumor growth. (a) Photographs of tumor before and after convection enhanced delivery of A5:ETE-PC followed by IRE. Images were taken 24-48 h post IRE. **(b)** Line graphs representing the growth of individual tumors in control and IRE groups. **(c)** Tumors were excised, weighed, and analyzed statistically. The bar graph represents mean tumor weight ± SEM, n=11. Unpaired t-test was used to compare difference in tumor weight; *p < 0.05

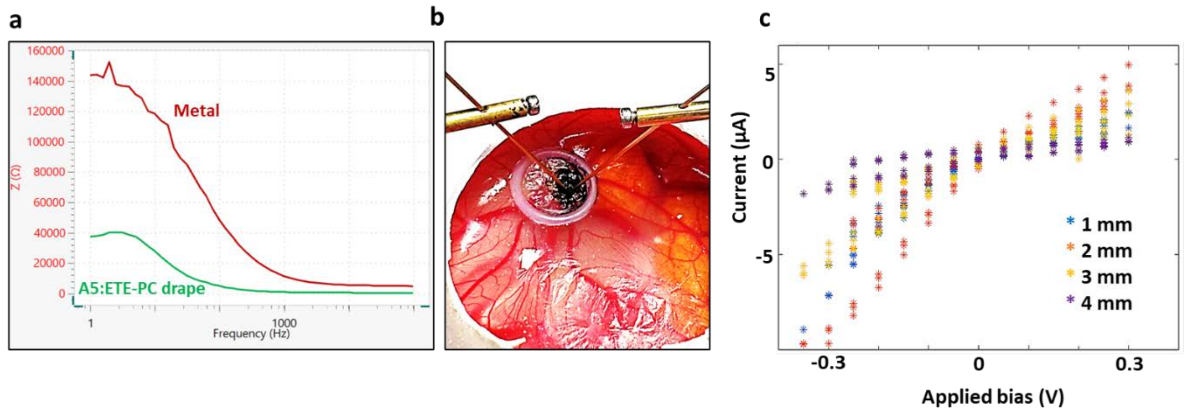

Fig. 15: EIS analysis of A5:ETE-PC Drape (a) EIS analysis if tumor cavity before and after drape formation. Bode plot represents impedance of metal electrode vs A5:ETE-PC drape in resection cavity. **(b)** Electrical analysis was performed between two Au coated electrodes placed at various distances on A5:ETE-PC drape in resection cavity. **(c)** Current–voltage sweeps obtained between the contacts on drape shown in the figure.

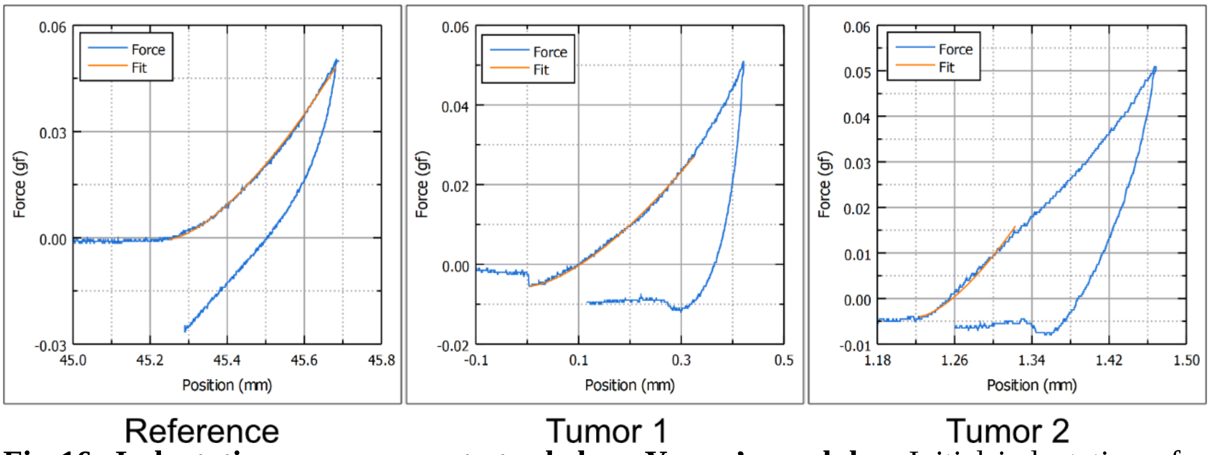

Fig 16: Indentation measurements to deduce Young's modulus. Initial indentation of a pristine tumor cavity sample (reference) and two drape covered tumor resections (tumor 1/2). The red lines show the Hertzian fit as given by the Biomomentum software. Please note that the curves do not return to 0 force, suggesting adhesion. Young's moduli were estimated assuming about 7mm thickness and 0.45 for the Poisson ratio.

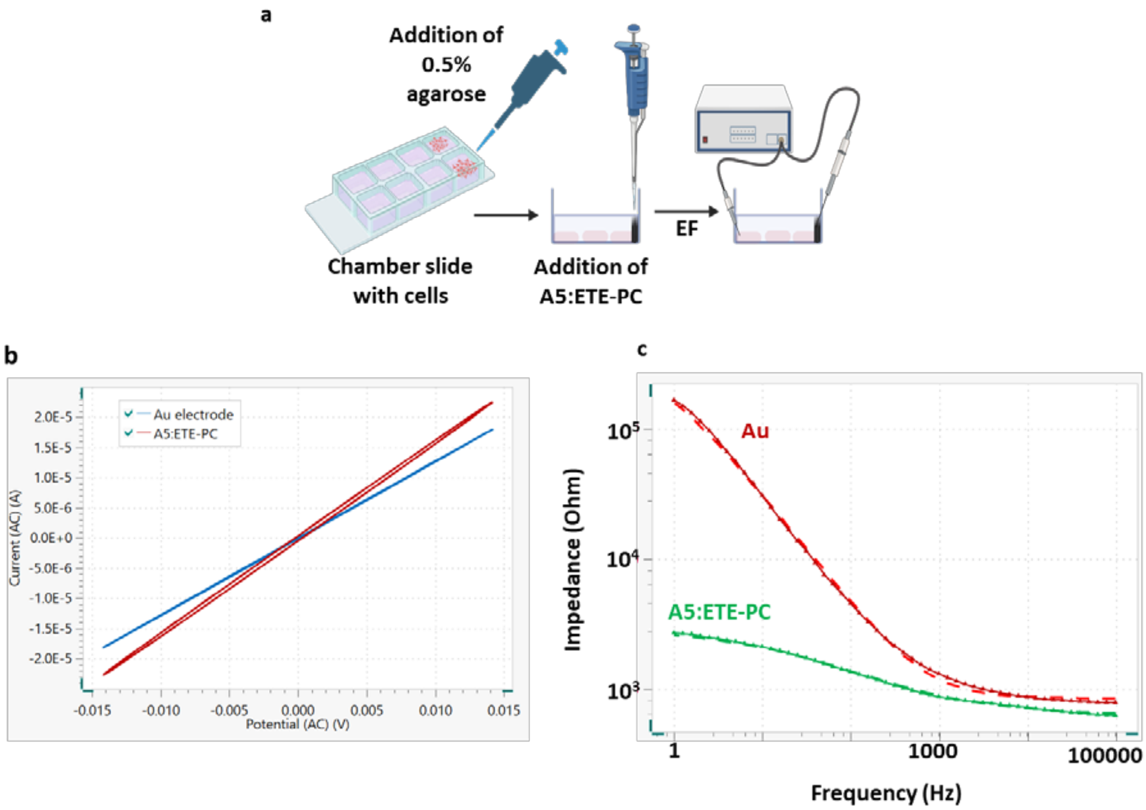

Fig. 17: Evaluation of in vitro model for cell-based studies. (a) Schematic representation of A5:ETE-PC electrode assembly for in vitro studies. **(b)** Lissajous plot showing current as a function of potential for EIS measurements using Au coated metal or A5:ETE-PC electrodes in in vitro agarose model for cell-based studies. **(c)** Bode plots showing impedance measurement using Au coated metal or A5:ETE-PC electrodes in in vitro agarose model for cell-based studies. **Figure 17a** created with BioRender.com released under a Creative Commons Attribution-NonCommcercial-NoDerivs 4.0 International license (*https://BioRender.com/8864ndt*).

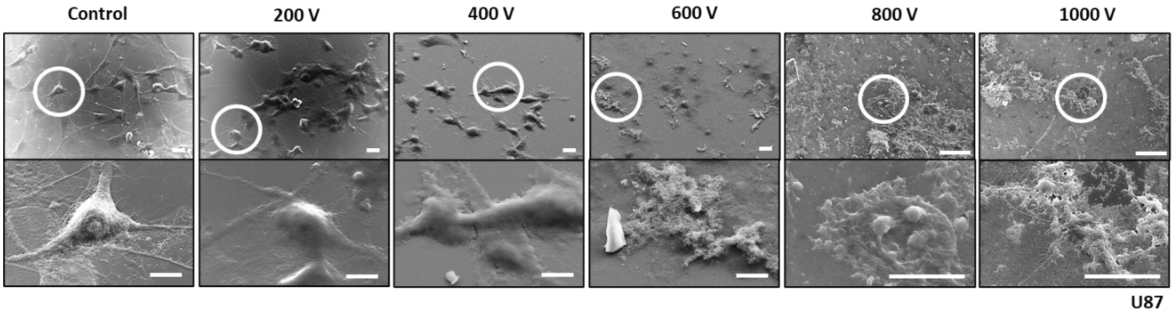

Fig. 18: A5:ETE-PC electrode induces irreversible electroporation in U87 cells. SEM images detailing the morphology of U87 cells undergone IRE; scale bar: 20 µm (upper panel), 10 µm (lower panel).

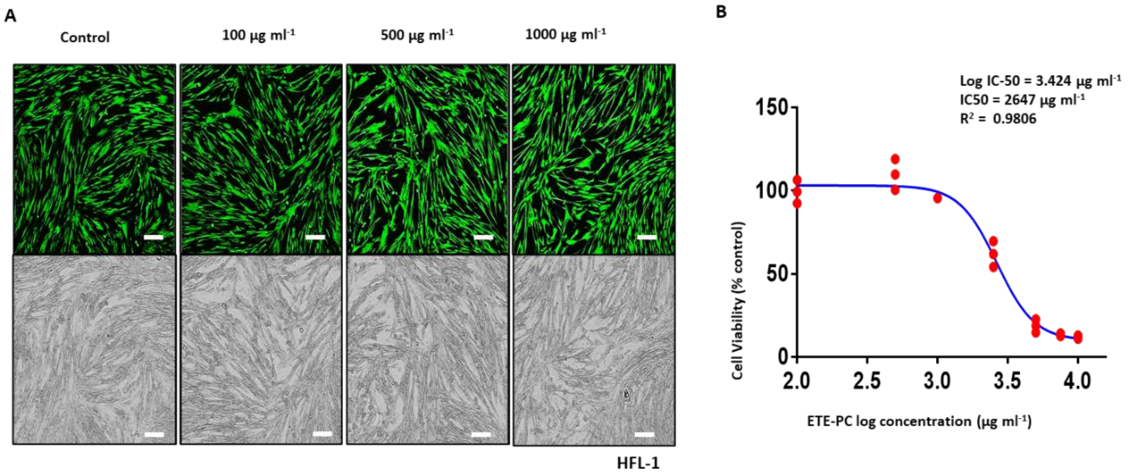

Fig. 19: In vtrobiocompatibility of ETE-PC. (a) Cytotoxicity of ETE-PC in normal human lung fibroblasts (HFL1) was determined by live(green)/dead(red) cell staining (Calcein-AM/PI) post-treatment with various concentrations of ETE-PC for 24 h. The upper and lower panels show fluorescence and corresponding brightfield images; scale bar: 100 µm. n=3 independent experiments**. (b)** Dose (log concentration) vs response curve showing IC50 of ETE-PC in HFL1 cells. The half-maximal inhibitory concentration (IC50) of ETE-PC in HFL1 cells was calculated by MTT cytotoxicity assay after treatment with 0.1–10 mg ml^{-1} of ETE-PC. n=3 independent experiments.

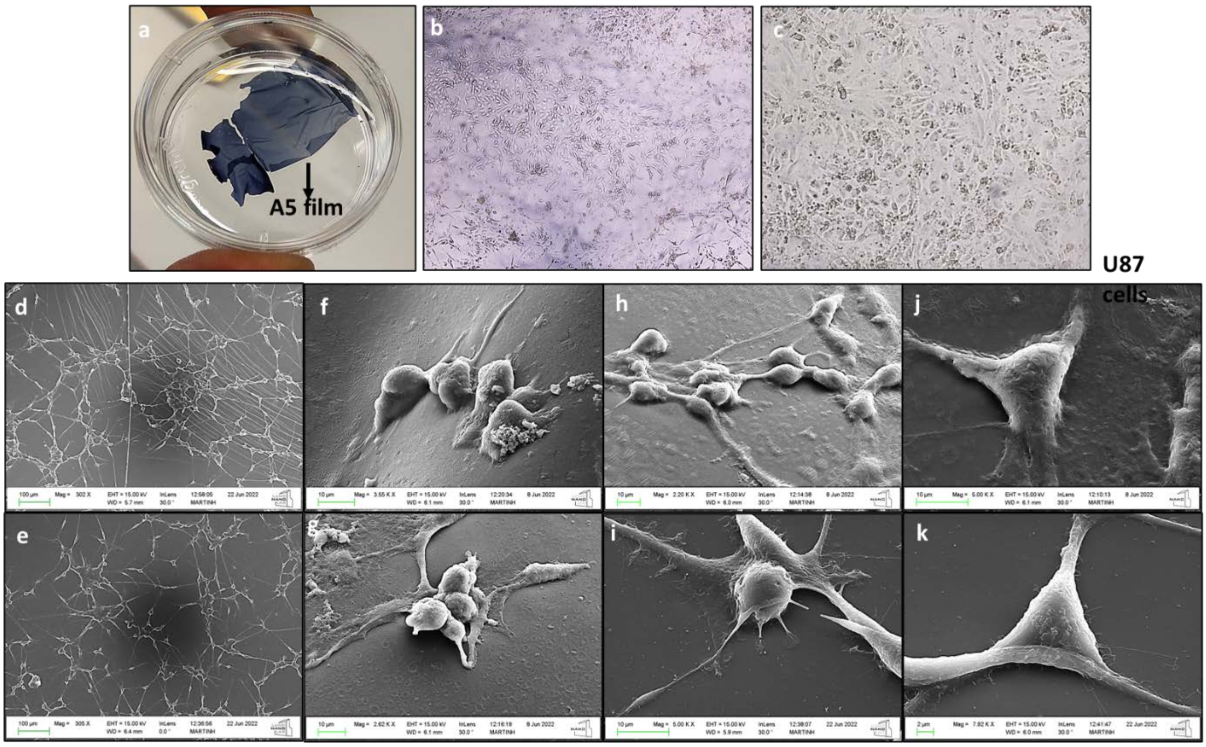

Fig. 20: Cell interaction with A5. (a) Image representing A5 film with U87 cells grown on it. **(b&c)** Phase contrast images of U87 cells grown on A5 film. **(d-k)** SEM images of U87 cells grown on A5 film. Scale bar denotes in **d, e** - 100 µm, **f-j** – 10 µm and **k** – 2 µm.

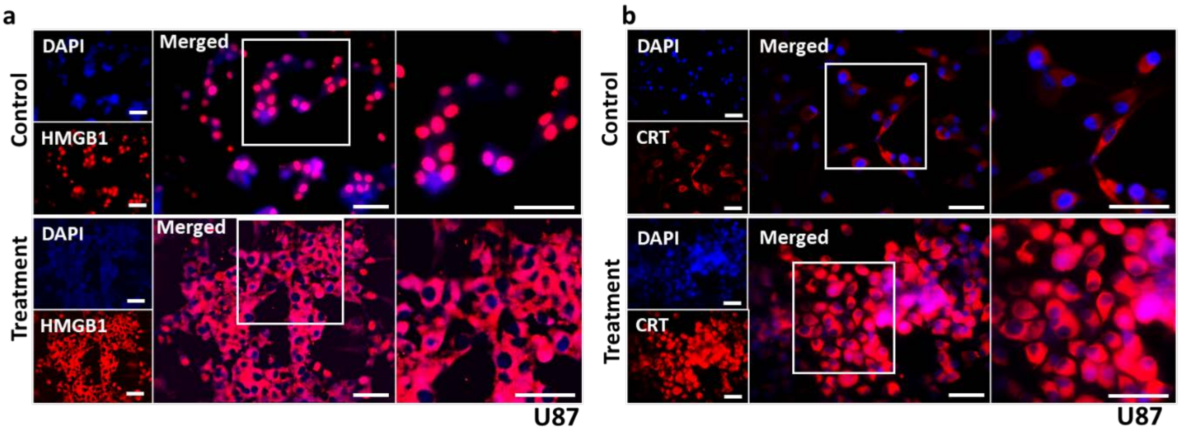

Fig. 21: **A5:ETE-PC mediated IRE induces immunogenic cell death (ICD).** Immunofluorescence images represent staining of ICD markers **(a)** HMGB1 and (**b)** calreticulin in U87 cells post-IRE via A5:ETE-PC electrode. Untreated cells were used as control. Scale bar: 100 µm. n=3 independent experiments.

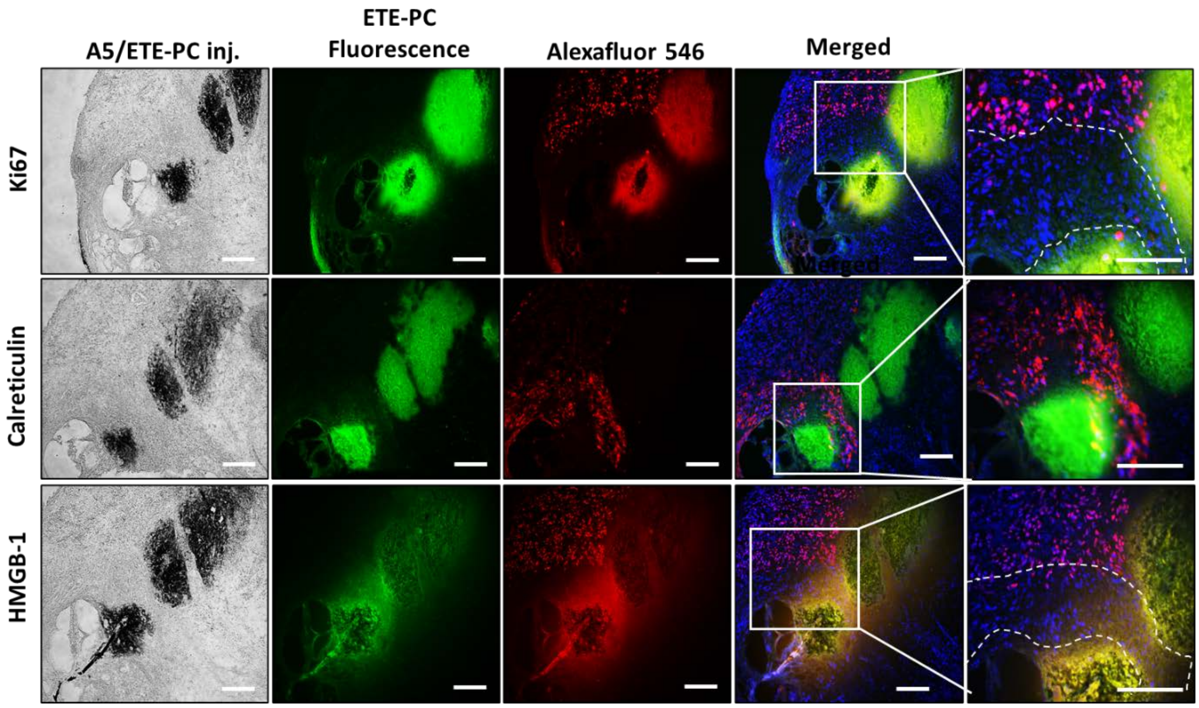

Fig. 22: IRE application via A5:ETE-PC electrode inhibits cell proliferation and induces immunogenic cell death in in vivo tumors. In situ assembly of A5:ETE-PC electrode was done in in vivo U87 tumors and IRE treatment (3 x 10, 500 V pulses, 1ms) was given. Tumors were harvested after 24 h, sectioned, stained and imaged using fluorescence microscope. Fluorescence images demonstrate staining of proliferation marker Ki67 and ICD markers HMGB1 & calreticulin in U87 tumor sections (16 µm). ETE-PC monomer fluorescence (green), Ki67 (red) and nucleus (DAPI, blue). Scale bar: 200 µm; n=1.

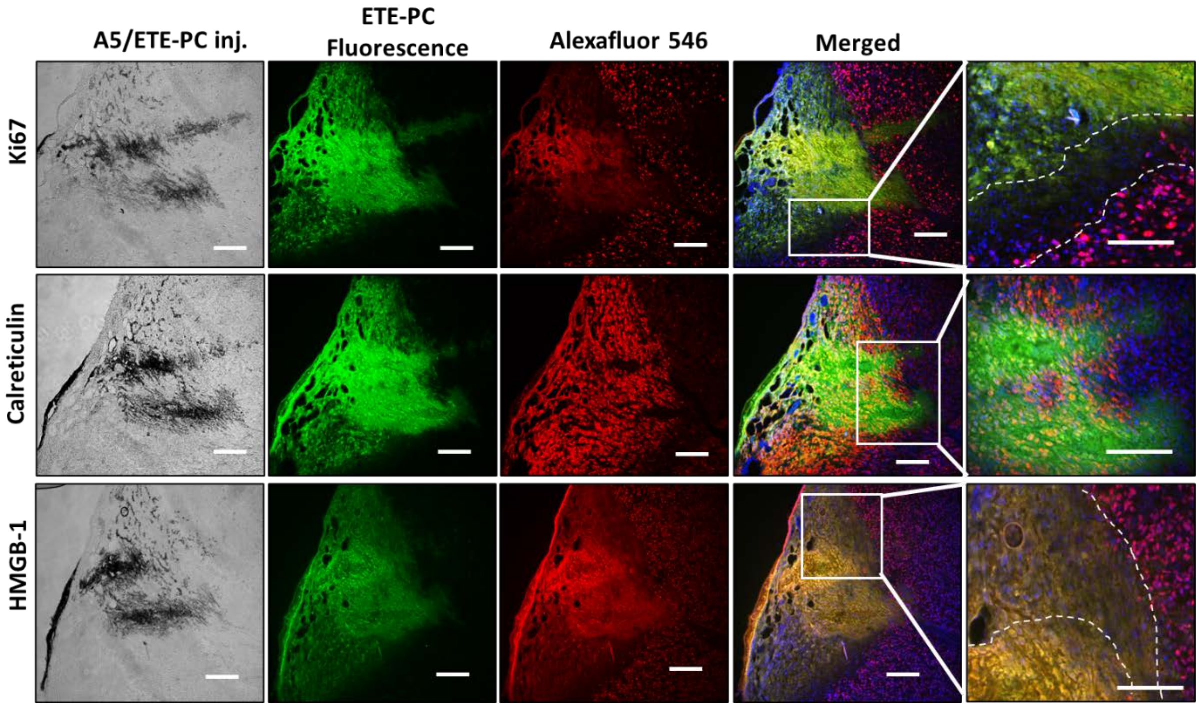

Fig. 23: IRE application via A5:ETE-PC electrode inhibits cell proliferation and induces immunogenic cell death in in vivo tumors. In situ assembly of A5:ETE-PC electrode was done in in vivo U87 tumors and IRE treatment (3 x 10, 2000 V pulses, 1ms) was given. Tumors were harvested after 24 h, sectioned, stained and imaged using fluorescence microscope. Fluorescence images demonstrate staining of proliferation marker Ki67 and ICD markers HMGB1 & calreticulin in U87 tumor sections (16 µm). ETE-PC monomer fluorescence (green), Ki67 (red) and nucleus (DAPI, blue). Scale bar: 200 µm; n=1.

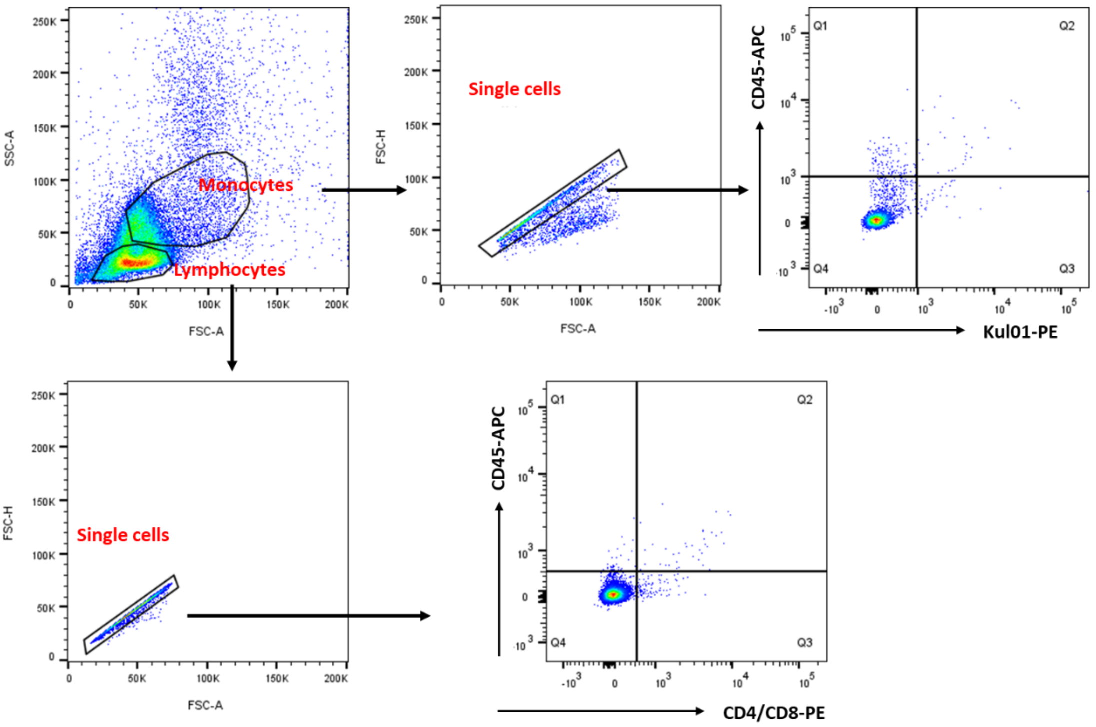

Fig. 24: Gating strategy for analysis of T lymphocytes and monocytes/macrophages.

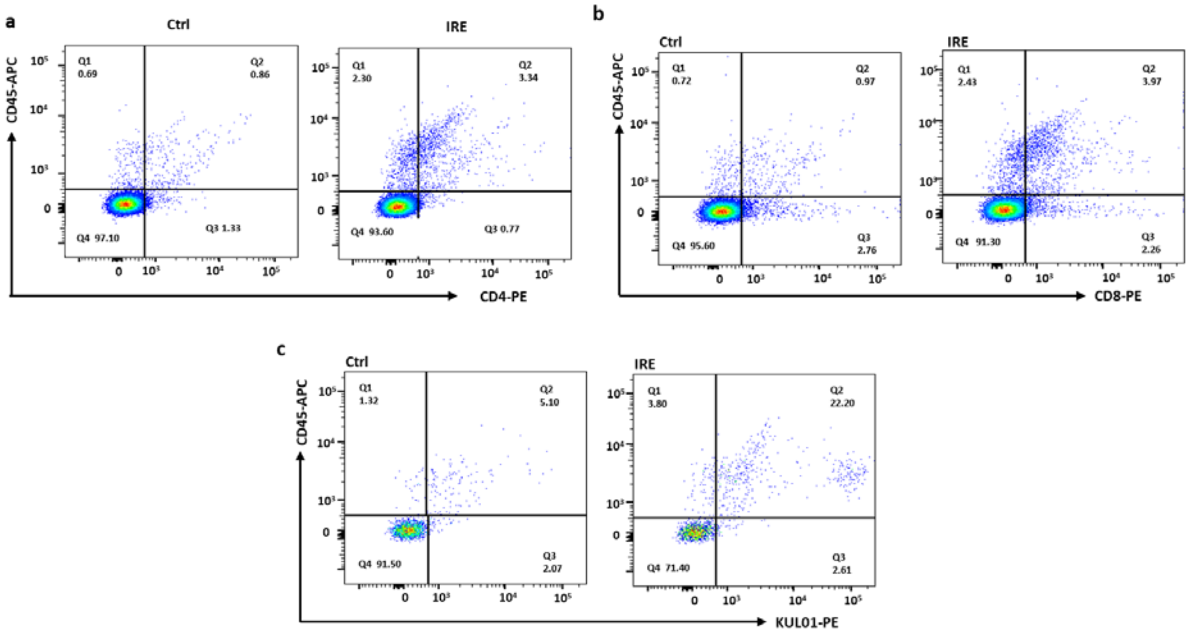

Fig. 25: IRE application via A5:ETE-PC electrode induces ICD and immune cell infiltration in tumors. (a-c) Immune cell infiltration was examined by flow cytometry as a percentage of $CD45^{+}CD4^{+}$ and $CD45^{+}CD8^{+}$ T cells along with $CD45^{+}KUL01^{+}$ macrophages in IRE treated (3 x 200, 1000-1500 V cm^{-1} pulses, 300 µs) vs control tumor 72 h post-Ire treatment.

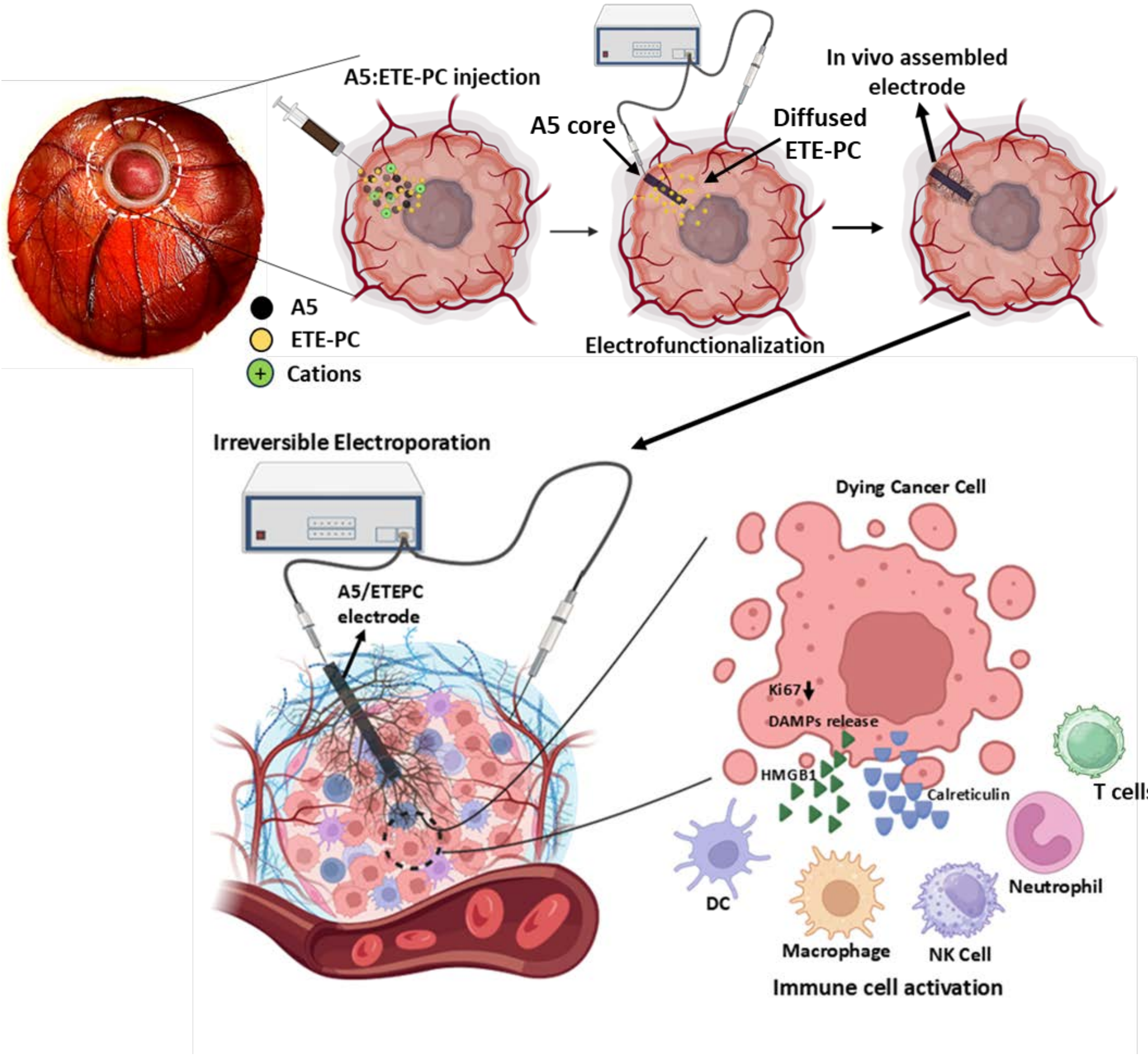

Fig. 26: A schematic representation of the in vivo electrode assembly and it's use for IRE in tumors. Figure created with BioRender.com released under a Creative Commons Attribution-NonCommcercial-NoDerivs 4.0 International license (*https://BioRender.com/8864ndt*).
